# Xpert MTB/RIF Ultra and mycobacterial culture in routine clinical care at a paediatric hospital

**DOI:** 10.4102/sajid.v37i1.398

**Published:** 2022-06-20

**Authors:** Anthony K. Enimil, James J.C. Nuttall, Chad M. Centner, Natalie Beylis, Brian S. Eley

**Affiliations:** 1 Department of Child Health, College of Health Sciences, Kwame Nkrumah University of Science and Technology, Kumasi, Ghana; 2 Department of Child Health, Komfo Anokye Teaching Hospital, Kumasi, Ghana; 3 Paediatric Infectious Diseases Unit, Red Cross War Memorial Children’s Hospital, Cape Town, South Africa; 4 Department of Paediatrics and Child Health, Faculty of Health Sciences, University of Cape Town, Cape Town, South Africa; 5 Division of Medical Microbiology, Faculty of Health Sciences, University of Cape Town, Cape Town, South Africa; 6 Department of Microbiology, National Health Laboratory Service, Groote Schuur Hospital, Cape Town, South Africa

**Keywords:** diagnosing childhood tuberculosis, respiratory specimen, Xpert MTB/RIF Ultra, mycobacterial culture, incremental yield, microcytic anaemia

## Abstract

**Background:**

Microbiological confirmation of pulmonary tuberculosis (PTB) in children is a well-documented challenge. This study evaluated Xpert Mycobacterium Tuberculosis (MTB)/Rifampicin (RIF) Ultra (Ultra) and mycobacterial cultures in routine clinical care at a tertiary paediatric hospital.

**Methods:**

Children treated for PTB and who had at least one respiratory specimen investigated by Ultra and mycobacterial culture before tuberculosis (TB) treatment was commenced were included. The findings of this retrospective study were summarised using descriptive and inferential statistics.

**Results:**

A total of 174 children were included. The median age was 2.5 years. Microcytic anaemia, airway compression, cavitary disease and miliary TB were significantly observed in children with microbiologically confirmed TB (cTB). Tuberculosis was microbiologically confirmed in 93 (53.4%) children. The positive yield from testing the first respiratory specimens was 68/174 (39.1%) on Ultra and 82/174 (47.1%) on combined Ultra and mycobacterial culture. In the subset of children (*n* = 70) tested with Ultra on two sequential respiratory specimens, the incremental yield from the second specimen was 30.3%. In the subset of children (*n* = 16) tested with Ultra on three sequential respiratory specimens, the incremental yield from the second and third specimens was 16.7% and 0.0%, respectively. When Ultra and mycobacterial culture results were combined, the incremental yield in children who had two sequential respiratory specimens tested was 24.4% and 3.1% on Ultra and mycobacterial culture, respectively.

**Conclusion:**

Ultra and mycobacterial culture on a single respiratory specimen resulted in a high microbiological yield. Ultra-testing on a second respiratory specimen increased the yield of microbiologically cTB. Additional diagnostic testing may require further study.

## Introduction

Globally, in 2020 there were approximately 10 million new cases of tuberculosis (TB), 12% of which were children. The global TB incidence rate was 130 new cases per 100 000 population per annum for all ages. South Africa is acknowledged as a high-burden country for TB, HIV-TB coinfection and multidrug-resistant TB (MDR-TB). In 2019 it had one of the highest incidence rates at 615 new TB cases per 100 000 population per annum with 360 000 incident cases.^1^

Sputum smear microscopy is widely used to detect TB. It has a number of limitations, including low sensitivity, especially in HIV-infected individuals and children, and the inability to detect drug-resistant TB.^2^ The Xpert MTB/RIF assay (Cepheid, Sunnyvale, CA, United States [US]) (Xpert) was endorsed by the World Health Organization (WHO) in December 2010 as a replacement for sputum smear microscopy, particularly in settings with high rates of HIV-associated TB and MDR-TB.^3^ It can detect *Mycobacterium tuberculosis* complex (MTBc) and simultaneously screens the β subunit of the bacterial RNA polymerase gene for the presence of mutations conferring rifampicin resistance.^4^

South African National Tuberculosis Control Programme introduced Xpert into clinical practice in March 2011.^5^ Prior to this intervention, confirmation of pulmonary TB (PTB) in children was carried out by smear microscopy and mycobacterial culture. The sensitivity of Xpert is superior to that of microscopy in children under investigation for TB.^6,7,8^ However, because childhood TB is paucibacillary, mycobacterial culture is superior to Xpert for diagnosing PTB in children.^9^ Thus, Xpert is used in combination with mycobacterial culture when investigating children for PTB at hospitals in South Africa.

An important paediatric TB diagnostic challenge is that most children are unable to produce an expectorated sputum (ES) specimen. Instead, alternative specimen types, notably induced sputum (IS) and gastric lavage (GL) aspirates, are used to investigate children with suspected PTB. The diagnostic performance of Xpert is comparable in IS and GL specimens.^10^

The sensitivity of Xpert may be increased by screening two or more sequential respiratory specimens from children under investigation for PTB.^6,11^ More recently, the Xpert MTB/RIF Ultra (Ultra) assay was developed to overcome the limited sensitivity of Xpert in the detection of PTB, particularly in patients with paucibacillary disease or HIV infection.^12^ The diagnostic performance of Ultra for confirming PTB in children was evaluated using banked sputum specimens obtained from children previously investigated for PTB. Compared with mycobacterial culture, the sensitivity and specificity of Ultra were 75.3% and 95.0%, respectively. In a subset of children, the sensitivity of Ultra was superior to that of Xpert, but mycobacterial culture outperformed both Xpert and Ultra. Furthermore, Ultra was unable to detect MTBc in specimens of 25.0% of children with culture-confirmed TB and hence cannot be used as a replacement test for mycobacterial culture, especially in settings where mycobacterial culture is part of the routine diagnostic workup.^13^

In February 2018, Ultra replaced Xpert as a diagnostic tool for TB in children at Red Cross War Memorial Children’s Hospital (RCWMCH), Cape Town. In this study, the microbiological status and the clinical and radiological features of TB in children at RCWMCH during the first year after the introduction of Ultra has been described. We evaluated the routine use of Ultra and mycobacterial culture in the diagnostic workup of these children.

## Methods

### Study design and setting

This retrospective study was conducted at RCWMCH in children treated for PTB and who had at least one respiratory specimen investigated by Ultra and mycobacterial culture before TB treatment was commenced. Red Cross War Memorial Children’s Hospital in Cape Town, South Africa is a 282-bed teaching hospital of the University of Cape Town. It serves as a tertiary-level paediatric referral hospital for sick children aged 0 to 13 years from the Western Cape province and surrounding provinces.

### Study population

Children aged 0 to 13 years who were investigated for TB and initiated treatment for microbiologically confirmed or unconfirmed PTB between 01 February 2018 and 31 January 2019 were included. These children were identified from the RCWMCH pharmacy and National Health Laboratory Service (NHLS) microbiology databases.

Inclusion Criteria:

- Children between 0 and 13 years who were treated for PTB, including those with concomitant extra-pulmonary tuberculosis (EPTB) at RCWMCH from 01 February 2018 to 31 January 2019
- Children who had Ultra ± mycobacterial culture results for at least one respiratory specimen recorded in the NHLS microbiology database.

Exclusion criteria:

- Children with EPTB without evidence of PTB
- Children who initially commenced TB treatment during the study period but then had their TB treatment stopped as they had an alternative diagnosis
- Children with PTB who started TB treatment before 01 February 2018.

The proportion of children with microbiologically unconfirmed TB (uTB) who met a consensus case definition of uTB as defined by an international expert committee was determined.^14^

### Data collection

Relevant demographic, clinical information and tuberculin skin test results were abstracted from paper-based medical records. Baseline chest radiographic findings were obtained from the radiology digital imaging database. Chest radiographs were viewed in both anterior-posterior or posterior-anterior (age-dependent) and lateral views. Relevant pathology was determined based on the integration of the radiologist’s report and researcher’s interpretation and recorded on a standardised radiology results sheet.^15^ Ultra and mycobacterial culture results and selective haematology and HIV results were abstracted from the NHLS electronic databases. All information was entered on study-specific data collection forms.

### Tuberculin skin testing

Tuberculin skin testing was performed by the Mantoux method. Briefly, 0.1 mL of 2 tuberculin units of purified protein derivative (Tuberculin PPD RT 23, 2 TU, AJVaccines, Copenhagen, Denmark) was administered intradermally with a short bevel needle. The extent of induration was measured after 48 h - 72 h. The result was interpreted according to WHO guidelines.^16^

### Study definitions

Confirmed TB (cTB) was defined as microbiological confirmation of MTBc by either culture or Ultra on at least one respiratory specimen. Unconfirmed TB was diagnosed if the attending clinician treated a child for TB based on the presence of suggestive symptoms or signs of TB, chest radiograph consistent with TB, a close TB contact and a positive tuberculin skin test but without microbiological confirmation.^14^

For the rest of the study definitions, see Online Appendix 1.

### Respiratory specimen collection/microbiological procedures

Respiratory specimens were collected by standardised IS, GL, expectoration, tracheal aspiration and bronchoalveolar lavage (BAL) methods.

Respiratory specimens were processed and analysed according to the laboratory’s standard operating procedures and relevant manufacturers’ instructions. Each specimen was processed for both Ultra and microbiological culture.

Refer to Online Appendix 1 for further details on specimen collection and microbiological procedures.

### Statistical analysis

All data were entered anonymously into a Microsoft Excel spreadsheet and exported to R statistical software version 3.5.1 for statistical analysis.^17^

Demographic, clinical and radiological categorical variables were presented as proportions and percentages of the total. Chi-squared test or Fisher’s exact test was used to assess the association of categorical variables between confirmed PTB and unconfirmed PTB. Fisher’s exact test was used when categorical variables were small values (5 and below).

Normally distributed continuous variables were summarised by the mean and standard deviation (s.d.). Student’s *t*-test was used to compare mean values of normally distributed variables in children with cTB and uTB. Skewed continuous variables were summarised by the median and interquartile range (IQR). The Wilcoxon rank-sum test was used to compare medians of non-normally distributed continuous variables in children with cTB and uTB. Statistical significance was set at *p* < 0.05.

All participants had at least one respiratory specimen for Ultra and mycobacterial culture. The microbiological yield was reported as number (%). For children with two or three specimens processed by Ultra and mycobacterial culture, the incremental yield between the first and second specimens and between the second and third specimens was calculated according to the method of Rachow et al.^11^

### Ethical considerations

Ethical approval was obtained from the Human Research Ethics Committee, Faculty of Health Sciences, University of Cape Town, reference number: HREC REF 049/2019. The RCWMCH Research Committee approved the study, reference number: RCC 177/2019. The study was conducted in accordance with the principles of the Declaration of Helsinki.

## Results

### Study participants and respiratory specimens

**FIGURE 1:**
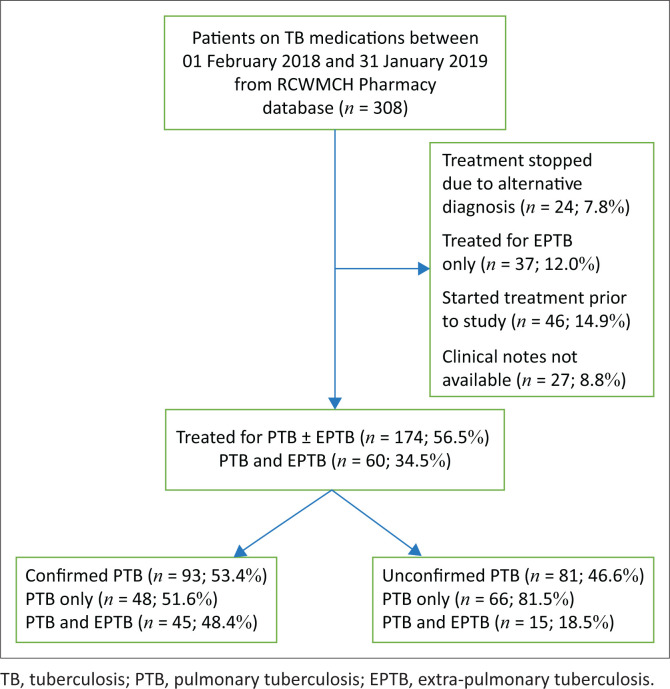
Selection of children with confirmed and unconfirmed pulmonary tuberculosis for data analysis.

During the study period, 308 children at RCWMCH received TB treatment, of whom 174 (56.5%) initiated TB treatment for PTB ± EPTB and were included in the analysis (Figure 1).

A total of 260 respiratory specimens were submitted for Ultra and mycobacterial culture in the 174 children. Specimen types were IS: 176 (67.7%) specimens from 126 children (72.4%); ES: 42 (16.1%) specimens from 24 children (13.8%); tracheal aspirate (TA): 25 (9.6%) specimens from 12 children (6.9%); GL: 14 (5.4%) from 11 children (6.3%); and BAL: three (1.2%) specimens from one child (0.6%).

A total of 70 children each had two respiratory specimens collected, a median of two days between the first and second specimens. A total of 16 children each had three respiratory specimens collected. The median interval between second and third specimens was 3.5 days. Of the 70 patients who had two specimens collected, 18 (25.7%) had different first and second specimen types. Of the 16 patients with three specimens, two (12.5%) had different second and third specimen types.

The median (IQR) age in years of study participants was 7.7 (6.4–10.3) for ES specimens, 2.4 (0.8–3.8) for GL specimens, 2.2 (1–5.1) for IS specimens and 1.8 (1.1–2.7) for TA specimens. The BAL specimens were obtained from a 2.8-year-old child.

Pulmonary TB was microbiologically confirmed by Ultra or mycobacterial culture in 2/3 (66.7%) BAL specimens; 16/25 (64%) TA specimens; 14/42 (33.3%) ES specimens; 58/176 (33.0%) IS specimens and 3/14 (21.4%) GL specimens.

### Tuberculosis classification

Tuberculosis was microbiologically confirmed in 93 (53.4%) of the participants. The prevalence of clinical features, tuberculin skin test reactivity and PTB exposure history in the 81 children with uTB is summarised in Table 1. Of these 81 children, 37 (45.7%) had documentation of Mantoux testing, of which 31 (83.8%) were positive. Of the 31 children with a positive Mantoux reaction, eight (25.8%) met the consensus case definition of uTB. The six (16.2%) with Mantoux negative results all had at least two of TB clinical diagnostic criteria, chest radiographs suggestive of PTB or exposure to PTB; that is, all six met the consensus definition of uTB. Of the 44 (54.3%) patients without Mantoux results, 17 (38.6%) had at least two of TB clinical diagnostic criteria, chest radiographs suggestive of PTB or exposure to PTB, which is consistent with the consensus definition of uTB.

**TABLE 1:** Prevalence of manifestations in children with unconfirmed pulmonary tuberculosis.

Variable	Total number (*n* = 81; 100%)		95% CI
	*n*	%	
**Clinical signs/symptoms suggestive of TB**			
Persistent cough > 2 weeks	20	24.70	15.8–35.5
Weight-for-age Z-score of ≤ −2	24	29.60	20.0–40.8
Persistent (> 1 week) and unexplained fever (> 38 °C)	4	4.90	1.4–12.1
Persistent, unexplained lethargy/reduced playfulness, *n*/*N* (%) (*N* = 78)	3	3.80	0.80–10.8
**Chest radiograph findings suggestive of PTB (*N* = 78)**	55	70.50	58.2–79.5
**A positive Mantoux test**			
≥ 10 mm if HIV uninfected children (*N* = 30)	24	80.00	72.3–90.4
≥ 5 mm if HIV-infected or weight-for-age Z-score < −2 (*N* = 174) (*N* = 7)	7	100.00	64.5–100
**Exposure to an individual with PTB**	40	49.24	37.4–60.2
**Unconfirmed cases fulfilling clinical, radiological, positive Mantoux and PTB exposure history criteria**			
Paediatric cases fulfilling all four criteria	7	8.60	3.5–17.0
Paediatric cases fulfilling three criteria	31	38.30	27.7–49.7
Paediatric cases fulfilling two criteria	34	42.00	31.1–53.5
Paediatric cases fulfilling one criterion	9	11.10	5.2–20.0

TB, tuberculosis; PTB, pulmonary tuberculosis; *N*, total number; *n*, number of events within a group; CI, confidence interval.

### Characteristics of confirmed and unconfirmed tuberculosis cases

The characteristics of the children with cTB and uTB are summarised in Table 2. Most of the children were less than 5 years of age. Amongst the 93 cases of cTB, 55 (59.1%) were diagnosed by both Ultra and mycobacterial culture, 23 (24.7%) by Ultra alone and 15 (16.2%) by mycobacterial culture alone. A significantly higher proportion of children with cTB experienced EPTB, *p* = 0.001. Amongst the 45 cTB cases with EPTB, 23 (51.1%) had central nervous system (CNS) TB, including 11 with miliary TB, 13 (28.9%) had abdominal TB and nine (20.0%) had EPTB involving other organ systems. Amongst the 15 uTB cases with EPTB, nine (60.0%) had CNS TB including five with miliary TB, three (20.0%) had abdominal TB and three (20.0%) had EPTB involving other organ systems. Significantly higher proportions of children with cTB were treated for complicated or CNS TB, *p* = 0.001.

**TABLE 2:** Demographic and clinical characteristics of children with confirmed and unconfirmed pulmonary tuberculosis.

Variables	All children (*N* = 174)		Confirmed PTB (*N* = 93)		Unconfirmed PTB (*N* = 81)		*p*
	*n*	%	*n*	%	*n*	%	
**Gender**							
Female	93	53.4	49	52.7	44	54.3	0.830
Male	81	46.6	44	47.3	37	45.7	
**Age category**							
< 5 years	126	72.4	65	69.9	61	75.3	0.430
≥ 5 and < 14 years	48	27.6	28	30.1	20	24.7	
**Childhood immunisation status**							
Completed for age	135	77.6	71	76.3	64	79.0	0.670
Incomplete for age	39	22.4	22	23.7	17	21.0	
**Moderate or severe underweight for age (Z-score < −2)**	55	31.6	31	33.3	24	29.6	0.600
**HIV infected**	21	12.1	11	11.8	10	12.3	0.910
**History of TB contact**	73	41.9	33	35.4	40	49.4	0.060
Contacts with positive GeneXpert	51†	69.9	25‡	75.8	26§	65.0	0.320
**Disseminated disease (PTB + EPTB)**	60	34.5	45	48.4	15	18.5	0.001*
**Types of TB treatment regimen**							
Uncomplicated	56	32.2	17	18.3	39	48.2	ref
Complicated	81	46.5	48	51.6	33	40.7	0.001*
Central nervous system	32	18.4	23	24.7	9	11.1	0.001
Individualised	1	0.6	1	1.1	0	0.0	0.990
Liver friendly	4	2.3	4	4.3	0	0.0	0.980

Note: All children: median age = 2.5 years, IQR = 1.1–5.3; confirmed PTB: median age = 2.5 years, IQR = 0.92–6.50; unconfirmed PTB: median age = 2.4 years, IQR = 1.42–4.75; *p* = 0.900.

IQR, interquartile range; TB, tuberculosis; PTB, pulmonary tuberculosis; EPTB, extra-pulmonary tuberculosis; *N*, total number; *n*, number of events in a group.

* , Significance at *p* < 0.05.

† , *N* = 73;

‡ , *N* = 33;

§ , *N* = 40.

The mean (s.d.) haemoglobin, white cell count and platelet count were 9.5 (2.0) g/dL, 14.7 (7.3) × 10^9^/L and 494 (211) × 10^9^/L, respectively. There were no statistically significant differences when comparing these parameters in children with cTB and uTB. However, a greater proportion of children with cTB, 33/92 (35.9%) compared with those with uTB, 15/74 (20.3%) had microcytic anaemia, odds ratio 2.2, 95% confidence interval (1.1–4.5), *p* = 0.03.

### Radiological features

Table 3 summarises the chest radiographic findings of children with cTB and uTB. Higher proportions of children with cTB experienced airway compression, cavitary disease and miliary TB.

**TABLE 3:** Radiological features of confirmed and unconfirmed pulmonary tuberculosis.

Intrathoracic lesions	Total (*N* = 168)†		Confirmed PTB (*N* = 90)		Unconfirmed PTB (*N* = 78)		*p*
	*n*	%	*n*	%	*n*	%	
Airspace opacification	92	54.8	45	50.0	47	60.2	0.24
Cavitary disease	5	3.0	5	5.6	0	0.0	0.035*
Lymph nodal disease	74	42.5	41	45.6	33	42.3	0.67
Airway compression	25	14.9	18	20.0	7	0.0	0.040*
Pleural effusions	20	11.9	8	8.9	12	15.4	0.19
Miliary disease	16	9.5	11	12.2	5	6.4	0.014*

*N*, total number; *n*, number of events in a group; PTB, pulmonary tuberculosis.

* , Significance at *p* < 0.05.

† , Six participants did not have chest radiographs.

Of the 168 (96.5%) participants with chest radiograph reports, 72 (42.8%) were 0–2 years old, 50 (29.8%) were between 2 and 5 years of age and 46 (27.4%) were above five years of age. Nodal disease was found in 35 (48.6%), 20 (40%) and 20 (43.5%) of the participants aged 0–2 years, two to five years and above five years, respectively. Airspace opacification was found in 43 (59.7%), 24 (48%) and 25 (54.3%) participants aged 0–2 years, two to five years and above five years, respectively.

### Microbiologically confirmed tuberculosis

Table 4 summarises the results of the respiratory specimens of the children processed by Ultra. The sensitivity of Ultra on the first respiratory specimens was 39.1% (68/174). A total of 70 (40.0%) children had a second respiratory specimen screened by Ultra. The first respiratory specimens of these children yielded 23 positive Ultra results. The second specimens of these 70 children yielded a further 10 positive Ultra results, an incremental yield of 30.3%. A total of 16 (9.2%) children had three respiratory specimens screened by Ultra. There was no incremental yield from the third Ultra respiratory specimen.

**TABLE 4:** Microbiological yield when respiratory specimens were evaluated by Xpert MTB/RIF Ultra but not mycobacterial culture.

Patients grouped according to the number of respiratory specimens submitted	Number of children (*N* = 174)		Number (*N* = 78) of positive Ultra results on the 1st respiratory specimens		Additional number (*N* = 174) of positive Ultra results on the 2nd respiratory specimens		Additional number of positive Ultra results on the 3rd respiratory specimens	
	*n*	%	*n*	%	*n*	%	*n*	%
1 specimen	174	100.0	68	39.1	-	-	-	-
2 specimens	70	40.0	23	32.9	10	14.3	-	-
3 specimens	16	9.2	4	25.0	1	6.3	0	0.0

*N*, total number; Ultra, Xpert MTB/RIF Ultra.

Table 5 summarises the results of the respiratory specimens of the children processed by both Ultra and mycobacterial culture. The sensitivity of Ultra and mycobacterial culture on the first respiratory specimens was 47.1% (82/174). The first respiratory specimens yielded 44.3% (31/70) positive Ultra and mycobacterial culture results for children with two respiratory specimens. Testing by Ultra of the second respiratory specimens added 10 positive results, an incremental yield of 24.4%. Mycobacterial culture testing of second respiratory specimens added one positive result, an incremental yield of 3.1%. There was no incremental yield from the third respiratory specimen for children with three respiratory specimens.

**TABLE 5:** Microbiological yield when respiratory specimens were evaluated by both Xpert MTB/RIF Ultra and mycobacterial culture.

Patients grouped according to number of respiratory specimens submitted	Number of children (*N* = 174)		Number (*N* = 90) of positive Ultra and/or MC results on the 1st specimens		Additional number of positive Ultra results on the 2nd specimens		Additional number of positive MC results on the 2nd specimens		Additional number of positive Ultra results on the 3rd specimens		Additional number of positive MC results on the 3rd specimens	
	*n*	%	*n*	%	*n*	%	*n*	%	*n*	%	*n*	%
1 specimen	174	100	82	47.1	-	-	-	-	-	-	-	-
2 specimens	70	40.0	31	44.3	10	14.3	1	1.4	-	-	-	-
3 specimens	16	9.2	5	31.2	1	6.3	0	0.0	0	0.0	0	0.0

*N*, total number; Ultra, Xpert MTB/RIF Ultra; MC, mycobacterial culture.

## Discussion

This study retrospectively compared the demographic, clinical and radiological features of children with confirmed and unconfirmed PTB at RCWMCH during the first year after introducing Xpert MTB/RIF Ultra. It also reviewed criteria for microbiologically unconfirmed PTB at RCWMCH in relation to a consensus case definition of uTB defined by an international expert committee.^14^ Incremental microbiological yield was assessed on second and third Ultra and mycobacterial culture results.

A total of 174 children were included. The median age was 2.5 years. Microcytic anaemia, airway compression, cavitary disease and miliary TB were significantly observed in children with microbiologically cTB.

Combining Xpert MTB/RIF Ultra and mycobacterial culture resulted in increased yield. A second sequential Xpert MTB/RIF Ultra test increased microbiological yield.

The median age amongst study participants was 2.5 years. This was similar to previous paediatric PTB studies at RCWMCH.^18,19^ This was an expected finding because children under five years have less developed immune systems and therefore more easily progress from primary infection to TB disease in high-burden TB countries than older children.^20^ The HIV prevalence was 12.1% amongst children with PTB in this study. Two earlier prospective studies at RCWMCH that enrolled 452 and 195 participants reported an HIV prevalence of 24% and 16.4%, respectively, amongst PTB patients.^6,19^ The lower prevalence in our study is likely attributable to the impact of the prevention of mother-to-child transmission of HIV interventions implemented in South Africa.

The prevalence of moderate or severe underweight for age amongst participants was 31.6% in this study. Zar et al.^19^ and Nicol et al.^6^ reported weight-for-age Z-score < −2 prevalence of 24.6% and 35.2%, respectively, amongst their participants. In this study, the prevalence of a positive Mantoux reaction in HIV uninfected participants with uTB was 80%. Zar et al.^19^ and Nicol et al.^6^ recorded a positive Mantoux reaction prevalence of 65.0% and 52.0% amongst HIV-uninfected participants with uTB. This study’s higher Mantoux positive prevalence estimate of 80% may be partly because of the small sample size inflating this prevalence estimate. The prevalence of radiological changes suggestive of PTB amongst study participants with uTB was 70.5%, whilst Nicol et al.^6^ reported a similar prevalence of 68.0%.

In this study, the prevalence of microcytic anaemia in microbiologically cTB and uTB participants was 35.9% and 20.3%, respectively. In a prospective, cross-sectional study conducted at another tertiary referral hospital in Cape Town in 1999, the prevalence of microcytic anaemia in cTB and probable TB was 26.0% and 29.0%, respectively.^21^

Anaemia of chronic disease, normochromic, normocytic anaemia, may be caused by TB. However, microcytic anaemia is usually because of iron deficiency in our setting. Thus, despite not performing confirmatory iron studies, it is likely that our patients had iron deficiency anaemia. Factors associated with iron deficiency anaemia in children living in sub-Saharan Africa include grain-based diets rich in phytates and phenols that inhibit the absorption of non-haem iron and helminthic infections that cause chronic gastrointestinal blood loss. Inflammation caused by infection such as TB can also contribute to the development of microcytic anaemia through hepcidin-mediated impairment of iron absorption and utilisation.^22^

The relatively high prevalence in both studies suggests that microcytic anaemia is endemic amongst children with TB in Cape Town and should be routinely screened for in these children. If present, iron studies can be used to confirm iron deficiency anaemia for the administration of iron replacement therapy.

This study found 54.8% airspace opacification, 42.5% nodal disease and 14.9% airway compression amongst all children treated for TB. In a paediatric study completed in Mozambique, airspace opacification was also the predominant feature of PTB.^23^ In this study, airspace disease was more frequent than nodal disease in all age categories, whereas in a retrospective case record review in British Columbia, nodal disease was more prevalent than airspace opacification in young and older children.^24^ The reason for these differences is not clear, although HIV infection could have influenced the radiological findings; in this study, 12% of participants had HIV infection, whilst none of the participants had HIV infection in the British Columbia study.

Of the 174 participants with at least one respiratory specimen, the overall sensitivity of Ultra was 44.8% (78/174) on any respiratory specimen. In a prospective study that consecutively recruited 195 hospitalised patients with at least one nasopharyngeal aspirate (NPA) and at least one IS, the sensitivity of Ultra was 10.3% and 15.9% on NPA and IS, respectively.^19^ Whilst Zar et al.^19^ obtained NPA and IS specimens from each study participant, this study retrospectively analysed a mixture of respiratory specimens types (ES, GL, IS and BAL) collected during routine clinical care.

In this study, in the 70 participants with two Ultra results on any respiratory specimen, the incremental yield from the second Ultra test result was 30.3%. In the 16 participants with three Ultra results, the incremental yield from the third Ultra test result was 0.0%. Furthermore, when we combined Ultra and mycobacterial culture results, the incremental yield from the second respiratory specimen tested by Ultra and mycobacterial culture was 24.4% and 3.1%, respectively. Rachow et al.^11^ prospectively enrolled 164 patients with suspected TB. Each participant provided up to three sputum specimens for smear microscopy, Xpert and mycobacterial culture. An incremental yield on second specimens and most third specimens were obtained when smear microscopy, Xpert and mycobacterial culture results were analysed separately and when Xpert and mycobacterial culture results were combined.^11^ In this study, the absence of microbial yield from the third respiratory specimen was probably because of the small number enrolled in this study. Thus, larger studies are probably needed to determine whether there are advantages to testing more than two respiratory specimens in routine clinical practice, particularly in settings where Ultra and mycobacterial culture are routinely performed.

This study had limitations. Some clinical information was not documented or available at the time of reviewing the participants’ information. The sample size for participants with at least one respiratory specimen was small. The numbers of participants with second and third respiratory specimens were also limited. Respiratory specimen types were requested during routine clinical care and determined by the attending clinicians. Thus, whilst the results suggest that processing two specimens by Ultra and mycobacterial culture improved results, further studies are needed to confirm this observation and determine whether additional specimens should be tested in routine clinical practice.

## Conclusion

In the first year of the introduction of Ultra at RCWMCH, the median age of children investigated and treated for TB was 2.5 years. Children with microbiologically cTB were more likely to have microcytic anaemia than those with uTB. On chest radiograph, cavitary, airway compression and miliary TB disease were more common in children with Ultra and mycobacterial culture-positive results than in children with negative results. Combining Ultra and mycobacterial cultures on a single respiratory specimen improved the prevalence of confirmed PTB in routine clinical care at RCWMCH. Testing second respiratory specimens increased the microbiological yield. Thus, where resources exist, testing two sequential respiratory specimens by Ultra and mycobacterial culture can be used to improve microbiological confirmation in children with PTB.
